# Effects of Cu Content on the Corrosion Resistance of Cu_x_CoCrMoNi High-Entropy Alloy

**DOI:** 10.3390/ma19051017

**Published:** 2026-03-06

**Authors:** Yuhua Peng, Ying Wang, Hansheng Zhao, Shuobin Chen, Yuxuan Liu, Mao Zhang, Pan Gong, Zhigang Hu, Ming Ma

**Affiliations:** 1 College of Artificial Intelligence, Wuchang University of Technology, Wuhan 430223, China; pyh8682937@163.com; 2 School of Mechanical Engineering, Wuhan Polytechnic University, Wuhan 430023, China; ywang0515@163.com (Y.W.); 13006337697@163.com (S.C.); 18372836536@163.com (Y.L.); hzg@whpu.edu.cn (Z.H.); maming@whpu.edu.cn (M.M.); 3 State Key Laboratory of Materials Processing and Die & Mould Technology, School of Materials Science and Engineering, Huazhong University of Science and Technology, 1037 Luoyu Road, Wuhan 430074, China; zhangmao@hust.edu.cn (M.Z.); pangong@hust.edu.cn (P.G.)

**Keywords:** high-entropy alloy, Cu element content, passive films, corrosion behavior

## Abstract

In this study, the corrosion behavior of Cu_X_CoCrMoNi (x = 0.3, 0.6, 0.9) high-entropy alloys (HEAs) in 3.5% NaCl solution is systematically investigated. The alloy samples show a strong link between copper content and corrosion resistance. It is noteworthy that an increase in copper content promotes element segregation, resulting in an increase in corrosion current density from 2.138 × 10^−7^ μA/cm^2^ to 1.8989 × 10^−6^ μA/cm^2^ and a decrease in charge transfer resistance from 182.6 Ω·cm^2^ to 42.34 Ω·cm^2^. In addition, electrochemical experiments demonstrate that lowering the copper content in the alloys reduces the spread and depth of corrosion. All alloys exhibit n-type semiconductor behavior, with donor density increasing from 4.792 × 10^23^ cm^−3^ to 5.581 × 10^23^ cm^−3^ with increasing copper content. Notably, the passive film is characterized by the presence of Cr_2_O_3_ and Cu_2_O as its main constituents. As the copper content in the HEA increases, higher levels of copper oxides in the passive film inhibit the formation of chromium oxides. This degrades the passive film quality, thereby diminishing the overall corrosion resistance.

## 1. Introduction

The durability of key components in the marine industry is heavily influenced by ocean conditions. Ship hulls operating at sea face constant corrosion risks from saltwater spray, marine organisms, and environmental changes. This damage worsens significantly in areas with high salt concentrations in seawater, frequent temperature fluctuations, and strong tidal forces, which speed up the breakdown of materials [1,2,3]. With the ongoing growth of the maritime industry, there is a growing need for materials exhibiting superior corrosion resistance. Among corrosion-resistant materials, high-entropy alloys (HEAs) stand out. HEAs consist of five or more principal elements with nearly equiatomic concentrations. This unique multi-principal element configuration gives rise to a high-entropy structure, enabling exceptional properties, including outstanding corrosion resistance. As such, they are ideally suited for use in harsh and corrosive environments, such as in marine applications [4,5,6,7,8,9].

Based on CoCrNi, the continuous exploration and design of new HEAs with excellent corrosion resistance have been carried out in recent years. Modifying the composition and microstructure has been demonstrated to be an effective approach for enhancing their properties [10,11]. Tsau et al. [12] fabricated CrFeCoNiV_x_ (x = 0.5, 1) HEA by incorporating Fe and adjusting the V content in the CoCrNi alloy system, subsequently investigating their corrosion behavior in NaCl environments. The CrFeCoNiV_0.5_ alloy exhibits a low corrosion current density of 1.8 µA/cm^2^, demonstrating superior corrosion resistance in 0.5 M NaCl solution at 30 °C. Han et al. [13] examined the corrosion resistance of a CoNiFeCrMn high-entropy alloy (HEA) by alloying Fe and Mn with CoCrNi and modifying its microstructure. The coarse-grained CoCrFeMnNi high-entropy alloy (HEA) demonstrated exceptional corrosion resistance in a 3.5 wt.% NaCl solution, exhibiting a notably low corrosion current density of 0.14 µA·cm^−2^ and a relatively noble corrosion potential of −0.23 V. Cu and Mo have been widely utilized as alloying elements in HEAs [14,15,16,17], owing to their ability to expand compositional diversity and enable the systematic investigation of corrosion characteristics. Notably, research on the CuCoCrMoNi HEA system remains very limited to date, particularly regarding its corrosion behavior, which has received minimal scientific attention. This significant knowledge gap underscores the critical need for comprehensive studies on the corrosion mechanisms of this alloy system.

Zhao et al. [18] systematically synthesized AlFeNiMnCu_x_ (x = 0.5, 0.75, 1.0, 1.25) high-entropy alloys (HEAs) via vacuum arc melting to evaluate their corrosion behavior in 3.5 wt.% NaCl solution at ambient temperature. Among the investigated compositions, the AlFeNiMnCu HEA demonstrated exceptional corrosion resistance in 3.5 wt.% NaCl solution at room temperature, demonstrating a corrosion current density of 22.7 µA/cm^2^. Wang et al. [19] prepared the AlCrFeNi3Cu_x_ (x = 0, 0.2, …, 1.0) alloy, and the alloy samples were synthesized via vacuum arc melting under an argon atmosphere. The electrochemical experiments on these samples were conducted in a 3.5 wt.% NaCl solution at 5 °C. The alloy containing Cu_0.4_ demonstrated superior corrosion resistance, as evidenced by a low corrosion rate of 0.0201 mm/yr, a minimal corrosion current density i_corr_ of 135 nA/cm^2^, and the highest charge transfer resistance R_ct_ of 4.516 × 10^6^ Ω·cm^2^. Wang et al. [20] prepared Cu_x_CoCrMoNi high-entropy alloys with different copper contents (x = 0, 0.3, 0.6, 0.9) using the vacuum arc melting method, and their tribological behaviors under 5–15 N load were systematically studied. The sample with x = 0.3 demonstrated optimal corrosion resistance, exhibiting the lowest corrosion current density of 0.273 µA/cm^2^. These results confirm that Cu content is a critical factor in determining the corrosion resistance of these high-entropy alloys. However, research specifically addressing the corrosion behavior of CuCoCrMoNi system HEAs remains limited, with particularly sparse investigations systematically examining the influence of Cu content on their electrochemical properties. Therefore, this significant research gap highlights the necessity for comprehensive studies to expound the composition–property relationships in this alloy system.

This study systematically investigates the influence of copper content (x = 0.1, 0.3, 0.6) on the corrosion resistance of the Cu_x_CoCrMoNi high-entropy alloy (HEA) series in a 3.5 wt.% NaCl solution. The microstructural characteristics of the Cu_x_CoCrMoNi HEA were comprehensively analyzed through detailed characterization, including examinations of phase composition and elemental distribution. The corrosion behavior was analyzed using electrochemical experiments. The passive films formed on Cu_x_CoCrMoNi HEAs (x = 0.3, 0.6, 0.9) were characterized using Mott–Schottky analysis and X-ray photoelectron spectroscopy (XPS) to investigate their electronic properties and chemical composition, respectively.

## 2. Materials and Experimentation

### 2.1. Material Preparation

In this research, the vacuum arc melting technology prepares three sets of as-cast Cu_x_ (x = 0.3, 0.6, 0.9) CoCrMoNi HEAs. The purity of the single-metal elements is higher than 99.99%. All the alloy powders we used came from Zhongmai Metal Materials Co., Ltd., in Nangong City, China. The metal blocks were placed in a semicircular Cu mold from bottom to top in the order of their melting points, and the air in the furnace was withdrawn until the pressure dropped to 1 × 10^−3^ Pa. Afterward, the pure Ti ingot, previously placed in the center of the mold, was filled with Ar gas and melted in an Ar atmosphere to remove oxygen from the furnace altogether. To ascertain a uniform compound distribution, the alloy ingot underwent at least 5 remelting cycles within a hemispherical Cu mold. Subsequently, the ingots were meticulously cleaned using deionized water and an alcohol rinse. Subsequently, energy-dispersive X-ray spectroscopy (EDS) was employed to determine the bulk composition of the CuxCoCrMoNi HEAs (x = 0.3, 0.6, 0.9), with the results summarized in Table 1.

The phase structure of the Cu_x_CoCrMoNi HEAs (x = 0.3, 0.6, 0.9) was characterized by X-ray diffraction (XRD; XRD-7000, Shimadzu Corporation, Japan) using Cu Kα radiation (λ = 0.154 nm) at 40 kV and 100 mA. Diffraction patterns were recorded over a 2θ range of 20–100° at a scanning rate of 8°/min. Microstructural characterization, including secondary electron imaging and elemental mapping, was performed using a field emission scanning electron microscope (FE-SEM; Quanta 650FEG, FEI, Czech FEI Hong Kong Company, Hong Kong, China) equipped with an energy-dispersive X-ray spectroscopy (EDS) system, operated at an accelerating voltage of 20 kV. Microstructural characterization and corrosion testing are conducted on the surface of the sample.

### 2.2. Electrochemical Corrosion Test 

Samples of the Cu_x_CoCrMoNi HEAs (x = 0.3, 0.6, 0.9), with dimensions of 15 × 15 × 2 mm^3^, with a weight of 10 g, were sectioned from as-cast ingots via electrical discharge wire cutting. The specimens subsequently underwent sequential surface preparation through mechanical grinding with SiC abrasive paper, progressing from 400 to 2000 grit. Final mirror-polishing was achieved using a 2.5 µm diamond polishing compound.

Electrochemical measurements were performed using a CS310X workstation in a standard three-electrode cell, with the Cu_x_CoCrMoNi HEAs (x = 0.3, 0.6, 0.9) as the working electrodes, a platinum mesh as the counter electrode, and a saturated calomel electrode (SCE) as the reference. All tests were performed in a 3.5 wt.% NaCl aqueous solution at room temperature. Before testing, each working electrode was stabilized at a potential of −0.2 V vs. SCE for 120 s to remove surface contaminants. The open-circuit potential (OCP) was monitored for one hour to achieve a steady state. Electrochemical impedance spectroscopy (EIS) was then performed at the stabilized OCP with a sinusoidal amplitude of 10 mV over a frequency range of 10^5^ Hz to 10^−2^ Hz. Finally, potentiodynamic polarization curves were recorded by sweeping the potential from −0.5 V to +1.5 V vs. SCE at a scan rate of 0.5 mV/s.

To further investigate the passive film characteristics, Mott–Schottky analysis was conducted on the HEAs using a 10 mV signal with cathodic polarization at a cathodic direction with a 25 mV potential step. Following a cathodic pre-polarization from −2.5 V to 0 V (vs. SCE), a stable passive film was formed by applying a potentiostatic hold at +0.3 V (vs. SCE) for 1800 s, followed by XPS analysis to characterize their chemical compositions. All samples were tested in triplicate under identical conditions to ensure reliable and reproducible results. Following the electrochemical analyses, to precisely characterize surface topography, the post-polarization morphology of the HEA was examined with a high-resolution laser confocal microscope (S neox 090, SENSOFAR, Beijing Yiguang Technology Co., Ltd, Beijing, China).

The chemical composition of the passive film was analyzed by X-ray photoelectron spectroscopy (XPS; Axis-ultra DLD-600W, Shimadzu Kratos Corporation, Japan) using a monochromatic Al Kα source (hv = 1486.6 eV). All spectra were calibrated to the adventitious carbon C 1s peak at 284.8 eV and subsequently deconvoluted using the Advantage 2022 software (v5.9921, Thermo Fisher Scientific, USA).

## 3. Results and Discussion

### 3.1. Cu_x_CoCrMoNi HEA Microstructure Characterization

According to Hume–Rothery principles, the phase structure of an alloy system is determined primarily by inter-elemental chemical affinity. In high-entropy alloys, however, the substantial disparities in affinity strengths between the multiple principal elements give rise to a wide spectrum of possible phase formations. HEAs typically form simple phase structures when the *ΔH_mix_* ranges between −40 and 10 kJ/mol, and exhibit a preference for FCC phase formation when the VEC reaches 8 or higher [21,22]. Based on calculations, it is found that the VEC for the Cu_0.3_CoCrMoNi HEA is 8.21, the *∆H_mix_* is −0.0461 kJ/mol, and the *∆S_mix_* is 1.54R; for the Cu_0.6_CoCrMoNi HEA, the VEC is 8.39, *∆H_mix_* is 0.0202 kJ/mol, and *∆S_mix_* is 1.59R; for the Cu_0.9_CoCrMoNi HEA, the VEC is 8.55, *∆H_mix_* is 0.0836 kJ/mol, and *∆S_mix_* is 1.6R. The VEC values for all Cu_x_CoCrMoNi HEAs are observed to exceed 8, and the *∆H_mix_* values range from −40 to −6.45 kJ/mol. Consequently, the XRD analysis indicates that the Cu_x_CoCrMoNi HEAs are inclined to form an FCC phase structure, thus confirming the accuracy of the XRD analysis.

**Figure 1:**
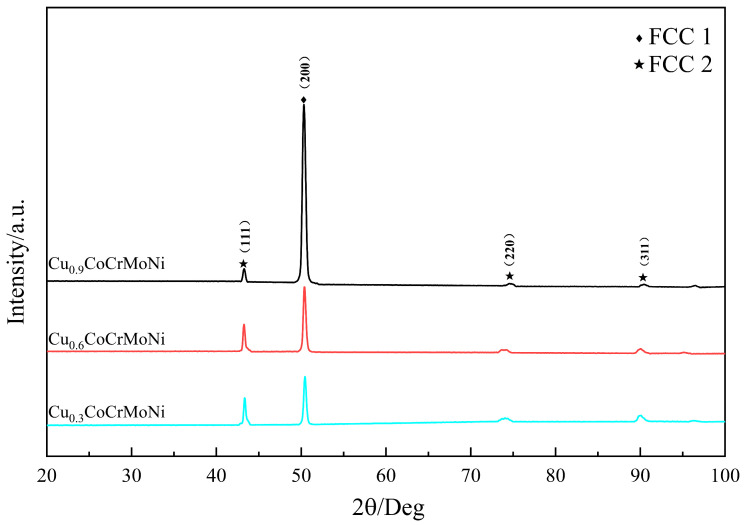
XRD images of different contents of Cu_x_ (x = 0.3, 0.6, 0.9) CoCrMoNi HEA; all the diffraction peaks are FCC1/FCC2 two-phase structures [20].

Figure 1 shows the XRD patterns of Cu_x_ (x = 0.3, 0.6, 0.9) CoCrMoNi HEA samples. We use a standard PDF card as the standard reference data for the qualitative analysis of X-ray diffraction phase, with the reference number ICDD PDF No. 01-079-0147. The results reveal that increasing Cu content does not alter the phase constituents of alloys and no new phases are generated. All three alloys are composed of FCC structures. Further analysis indicates that the intensity of FCC2 diffraction peak intensity increases in Cu_0.6_CoCrMoNi and Cu_0.9_CoCrMoNi compared to Cu_0.3_CoCrMoNi. When Cu content exceeds 0.6, the (200) peak at 50.3° shows significant enhancement. In contrast, the intensities of the (111) and (311) peaks are slightly reduced, and the analysis reveals that the proportion of the FCC2 phase increases progressively with the rising copper concentration while the FCC1 phase decreases. This evolution is primarily governed by the mixing enthalpy (*ΔH_mix_*) and the significant atomic size mismatch between copper and the other principal elements (Co, Cr, Mo, Ni), which lead to limited mutual solubility among these elements, resulting in compositional heterogeneity and segregation. [23]. These factors promote segregation and secondary phase formation during solidification.

**Figure 2:**
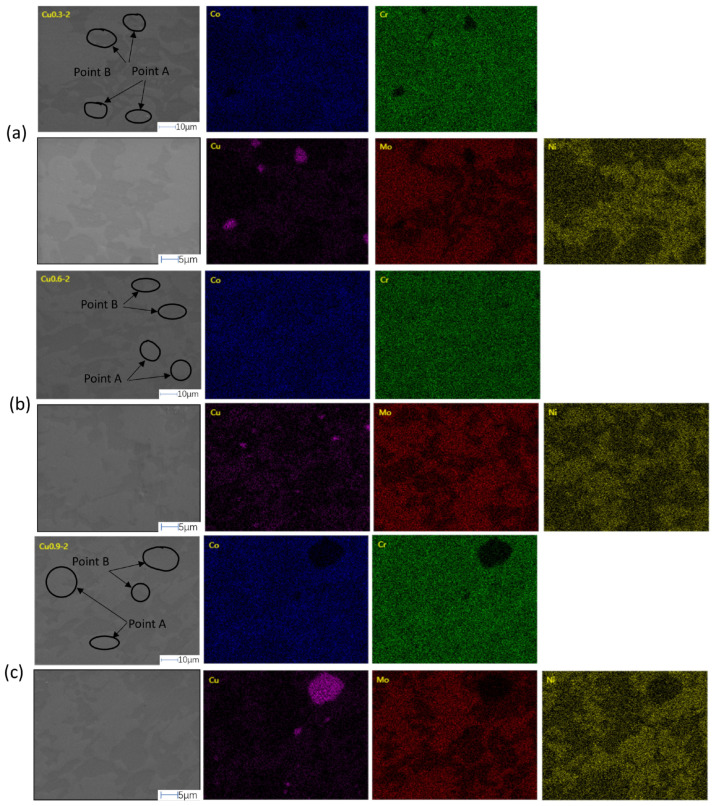
EDS point and surface swept images of Cu_x_ (x = 0.3, 0.6, 0.9) CoCrMoNi HEA: (**a**) Cu_0.3_CoCrMoNi, (**b**) Cu_0.6_CoCrMoNi, and (**c**) Cu_0.9_CoCrMoNi [20].

The SEM images of three sets of Cu_x_ (x = 0.3, 0.6, 0.9) CoCrMoNi HEA and EDS mapping are displayed in Figure 2. EDS point analyses are performed at points A and B in the red frame shown in the SEM images, and the resulting elemental compositions are tabulated in Table 2. The SEM images exhibit that three HEAs with different Cu contents have a typical two-phase structure. The white-gray areas (point A) and black areas (point B) can be observed. The morphology of these regions is shown to be elongated stripes or rounded from images. In addition, significant elemental segregation can be observed from the red frames in Figure 2.

The Cu element is prone to forming Cu-rich and nano-precipitated phases during solidification [24,25]. The EDS characterization results of points A and B show that Cu is rich at point B than at point A, indicating that Cu is more concentrated in black areas. Thus, the black regions correspond to a Cu-rich phase, whereas the white-gray regions represent a Cu-poor phase.

The analysis reveals that the Cu_x_CoCrMoNi HEAs (x = 0.3, 0.6, 0.9) possess a dual-phase characterized by the coexistence of FCC1 and FCC2, formed as a result of elemental segregation. This phase separation phenomenon demonstrates a significant compositional dependence, with the degree of elemental segregation progressively intensifying as Cu content increases. Within the series, the Cu_0.9_CoCrMoNi alloy exhibits the most pronounced elemental segregation, where the phase boundary distinction becomes particularly pronounced.

### 3.2. Corrosion Resistance of Cu_x_CoCrMoNi HEA in 3.5 wt.% NaCl Solution

**Figure 3:**
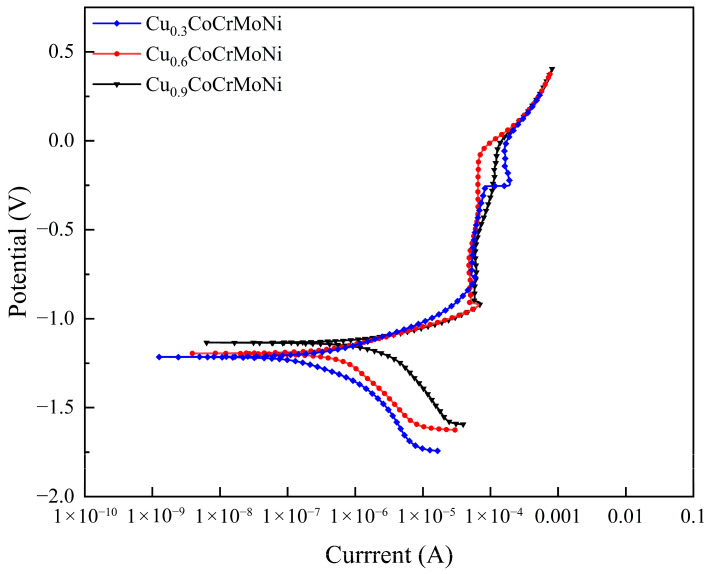
Potentiodynamic polarization curves for Cu_0.3_CoCrMoNi, Cu_0.6_CoCrMoNi, and Cu_0.9_CoCrMoNi HEA.

As shown in Figure 3, the potentiodynamic polarization curves of the Cu_0.3_, Cu_0.6_, and Cu_0.9_CoCrMoNi HEAs in 3.5 wt.% NaCl solution all demonstrate well-defined passive regions. This confirms the formation of stable passive films on these alloys under the given testing conditions [26]. Furthermore, the anodic Tafel curve exhibits an active–passive transition behavior with increasing applied potential evidencing the stability of the formed passive films, a characteristic closely linked to their protective efficacy in chloride-containing environments [27,28]. Table 3 summarizes the key electrochemical corrosion parameters obtained from Tafel analysis: corrosion current density (I_corr_), initial passive potential (E_pp_), and passive current density (I_pass_). In particular, the Cu_0.3_CoCrMoNi, Cu_0.6_CoCrMoNi, and Cu_0.9_CoCrMoNi alloys exhibited I_corr_ values of 2.138 × 10^−7^ μA/cm^2^, 5.0647 × 10^−7^ μA/cm^2^, and 1.8989 × 10^−6^ μA/cm^2^, respectively, demonstrating a progressive increase in corrosion current density with higher Cu content. The results revealed a positive correlation between Cu content and I_corr_, with the Cu_0.3_ alloy exhibiting the lowest corrosion current density and the Cu_0.9_ alloy showing the highest corrosion current density. These reduced I_corr_ values generally imply enhanced corrosion resistance and material stability, which effectively inhibits passive film dissolution in corrosive environments [29,30,31]. A quantitative evaluation of the integrated corrosion resistance for the Cu_x_CoCrMoNi HEA system was conducted, and the parameter E_pp_ was introduced to reflect the initial activation–passivation potential and to quantify the passivation kinetics through film formation rate analysis during electrochemical degradation [32,33]. As demonstrated in Table 3, the Cu_0.3_CoCrMoNi HEA exhibits significantly higher Epp values compared to both the Cu_0.6_CoCrMoNi and Cu_0.9_CoCrMoNi HEA samples. Thus, Cu_0.3_CoCrMoNi HEA possesses a lower corrosion current and a higher E_pp_ value than Cu_0.6_CoCrMoNi HEA and Cu_0.9_CoCrMoNi HEA. Comparative analysis identified the Cu_0.3_CoCrMoNi composition as possessing the optimal corrosion resistance, followed by the Cu_0.6_CoCrMoNi sample, while the Cu_0.9_CoCrMoNi alloy demonstrated the lowest corrosion resistance.

Therefore, with increasing Cu content in the alloys, the corrosion current density becomes progressively higher, which indicates that the alloys with higher Cu content exhibit an increasingly greater corrosion rate. These results demonstrate a clear inverse correlation between Cu content and corrosion resistance in the alloy system.

**Figure 4:**
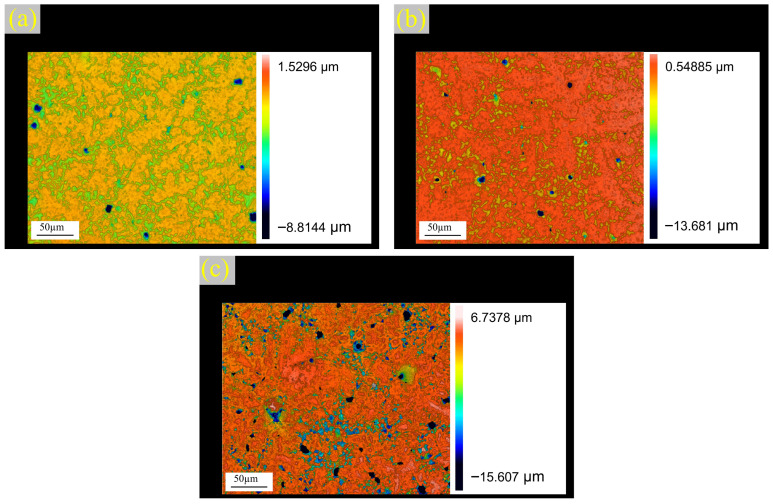
Three-dimensional images of Cu_x_ (x = 0.3, 0.6, 0.9) CoCrMoNi HEAs after polarization in 3.5 wt.% NaCl solution with (**a**) Cu_0.3_CoCrMoNi, (**b**) Cu_0.6_CoCrMoNi, and (**c**) Cu_0.9_CoCrMoNi.

The surface corrosion morphology of the Cu_x_CoCrMoNi HEAs after potentiodynamic polarization testing was examined using super-depth-of-field laser confocal microscopy, with the results shown in Figure 4. The maximum corrosion pit depths measured on the passive films were 8.81 µm for Cu_0.3_CoCrMoNi, 14.89 µm for Cu_0.6_CoCrMoNi, and 15.61 µm for Cu_0.9_CoCrMoNi. The surface of Cu_0.3_CoCrMoNi is relatively smooth, and only a few pitting pits are scattered. The surface of the Cu0.6CoCrMoNi sample exhibited corrosion pits that exhibit a substantially higher density and quantity, with both parameters progressively increasing with depth. For the Cu_0.9_CoCrMoNi sample surface, the diameter and the number of corrosion pits increase continuously. This is caused by further corrosion of the Cl^−^. The results show that the Cu_0.3_CoCrMoNi HEA sample has shallower depth and lower density of corrosion pits, suggesting that the Cu0.3CoCrMoNi HEA demonstrated superior corrosion resistance among the tested compositions. Corrosion pit depth is a critical parameter governing the corrosion resistance of HEAs [34].

Thus, with the increase in Cu content, the alloy exhibited more severe surface corrosion under corrosive conditions, characterized by wider distribution and deeper corrosion pits, indicating that lower Cu content contributes to enhanced corrosion resistance in the HEA.

**Figure 5:**
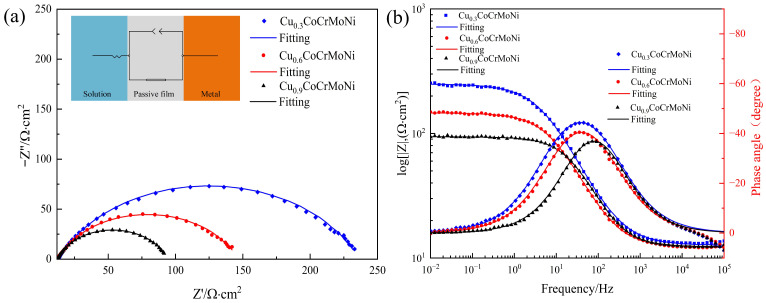
(**a**) Nyquist plots and (**b**) Bode plots and frequency–phase angle plots for Cu_x_ (x = 0.3, 0.6, 0.9) CoCrMoNi alloy (the inset of (**a**) shows the equivalent circuit for this measurement).

Figure 5 presents the electrochemical impedance spectroscopy (EIS) plots and their corresponding equivalent circuit fitting results for the Cu_x_CoCrMoNi HEAs with x = 0.3, 0.6, and 0.9. The capacitive semicircle diameters of the HEA samples follow a decreasing order, i.e., Cu_0.3_CoCrMoNi > Cu_0.6_CoCrMoNi > Cu_0.9_CoCrMoNi, as revealed by the analysis. An enlarged capacitive semicircle in the EIS spectrum indicates enhanced protection of the metallic substrate by the passive film against corrosive attack [35,36]. The corresponding Bode plots demonstrate three frequency-dependent regions in the HEAs: the Nyquist plots exhibit three distinct time constants, corresponding to high-, intermediate-, and low-frequency regions. The |Z| values of the three Cu_x_CoCrMoNi HEAs are approximately 12 Ωcm^2^ accompanied by near-zero phase angles. This behavior indicates predominant control by electrolyte solution resistance over the overall impedance response within this frequency range [37]. The intermediate-frequency region (10–10^3^ Hz) is characterized by a near-unity slope in the Bode magnitude plot and a well-defined peak in the phase angle plot. This behavior aligns with the characteristic signature of a system dominated by capacitive impedance within this spectral regime. At a low frequency, the |Z| values of 0–50 Ωcm^2^ suggest minimal charge transfer resistance, and Cu_0.3_CoCrMoNi exhibits optimal corrosion resistance, which is directly linked to the quality of its passive film. The EIS data indicate that this film acts as an effective diffusion barrier that isolates the substrate from the corrosive environment. As depicted in the Bode plot in Figure 5b, the Cu_0.3_CoCrMoNi HEA sample exhibits a significantly higher low-frequency impedance modulus |Z| compared to other samples, indicating enhanced corrosion resistance characteristics. As the frequency decreases, both Cu_0.6_CoCrMoNi and Cu_0.9_CoCrMoNi HEA samples consistently exhibit lower phase angles and notably narrower widths compared to the Cu_0.3_CoCrMoNi HEA sample. This indicates that Cu_0.3_CoCrMoNi HEA possesses a higher phase angle (−52°) with an extended plateau region. In general, the decreased phase angles observed in Cu_0.6_CoCrMoNi and Cu_0.9_CoCrMoNi HEAs suggest accelerated passive film dissolution due to enhanced charge accumulation at the alloy–electrolyte interface. This phenomenon correlates with reduced corrosion resistance in higher-Cu-content variants compared to the Cu_0.3_CoCrMoNi alloy.

The impedance spectra consistently exhibited a single capacitive time constant in the low-frequency region. Based on this observation, the electrical equivalent circuit (EEC) model shown in the inset of Figure 5a was employed to fit the EIS data [38,39]. In the equivalent circuit, Rs represents the resistance of the corrosive medium, while R_ct_ corresponds to the charge transfer resistance at the electrode/electrolyte interface. A constant phase element (CPE) is employed to model the non-ideal capacitive behavior of the passive film/electrolyte interface, thereby compensating for non-ideal interfacial effects and enabling a more accurate representation of the electrochemical system. The impedance is represented as follows [40]:

(1) $$Z_{CPE}=\frac{1}{Y_{0}{\left(j,\omega \right)}^{n}}$$

where *ω* is the angular frequency, j is an imaginary unit, Y_0_ is the scale factor, and n is the fitting index of the CPE (*n* = 1 denotes the CPE that is close to ideal capacitance; 1 < *n* < 0 means a pseudo-capacitance of semiconducting nature; *n* = 0 means a pure resistance) [41].

Table 4 lists the data calculated using the above equation. It can be observed that the R_ct_ values of the Cu_0.3_CoCrMoNi and Cu_0.6_CoCrMoNi HEA samples are 182.6 Ω·cm^2^ and 81.86 Ω·cm^2^, respectively. Moreover, the R_ct_ value of the Cu_0.9_CoCrMoNi HEA sample is 42.34 Ω·cm^2^. The R_ct_ of the HEA is progressively decreased with Cu increase. The charge transfer resistance (R_ct_), which directly reflects the corrosion resistance of the HEA, is inversely proportional to Cu content, with higher R_ct_ values corresponding to more protective passive films. The Z_CPE_ values can effectively reflect the defects of passive films. The n values for the three alloys are below 1, signifying defects on the passive film that appeared on HEA’s surfaces [42].

EIS analysis reveals a clear trend: the corrosion resistance of the Cu_x_CoCrMoNi HEAs in 3.5 wt.% NaCl solution deteriorates with increasing Cu content. This deterioration originates from Cu-induced destabilization of the protective passive film, where higher Cu concentrations exacerbate structural defects and chemical heterogeneity within the oxide layer, ultimately compromising its performance properties against chloride ion penetration.

In summary, as the copper content increases, the corrosion current density gradually increases, and the alloy shows more severe surface corrosion under corrosive conditions, characterized by a wider distribution and deeper corrosion pits. The increase in copper content disrupts the stability and integrity of the passivation film, leading to a significant decrease in the corrosion resistance of CuxCoCrMoNi high-entropy alloys in Cl^−^-containing environments.

### 3.3. Mott–Schottky Passive Film Performance Analysis

The corrosion resistance of HEAs is largely governed by the semiconductor properties of their passive films, as the corrosion rate is intrinsically linked to the electronic conductivity of these surface layers. To elucidate how Cu content modulates these properties, Mott–Schottky analysis was employed to determine the donor density of the passive films formed on the Cu_x_CoCrMoNi HEAs. The semiconductor properties of the passive film were analyzed using the Mott–Schottky relationship, which describes the dependence of the space-charge capacitance (C) on the applied potential (E) [43,44]:

For n-type semiconductors:

(2) $$\frac{1}{C^{2}}=\frac{2}{N_{D}e\varepsilon \varepsilon_{0}}(E-E_{FB}-\frac{KT}{e})$$

For p-type semiconductors:

(3) $$\frac{1}{C^{2}}=-\frac{2}{N_{A}e\varepsilon \varepsilon_{0}}(E-E_{FB}-\frac{KT}{e})$$

where ε is the dielectric constant of the passive film, ε = 15.6 for Cu_x_ (x = 0.3, 0.6, 0.9) CoCrMoNi HEA [45]; ε_0_ is the vacuum permittivity constant, with a specific value of about 8.85 × 10^−14^ F cm^−1^; e is the elementary charge (e = 1.602 × 10^−19^ c) [46,47]; N_d_ is the donor density; N_a_ is the acceptor density; E_FB_ is the flat band potential; T is the absolute temperature. N_d_ and Na can be derived from a straight-line slope fit to the Mott–Schottky curve.

**Figure 6:**
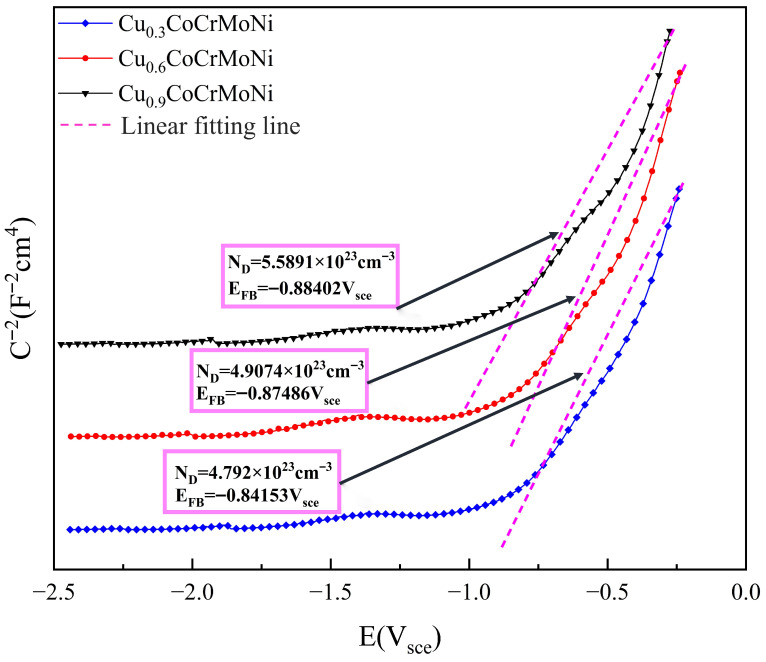
Mott–Schottky curves for Cu_0.3_CoCrMoNi, Cu_0.6_CoCrMoNi, and Cu_0.9_CoCrMoNi in 3.5 wt.% NaCl.

Figure 6 displays the Mott–Schottky plots obtained from the passive films of the Cu_x_CoCrMoNi HEAs with varying Cu content (x = 0.3, 0.6, 0.9). It demonstrates that the slopes of the curves of all three sets of three Cu-containing HEAs exhibit positive slopes in their Mott–Schottky curves under applied potentials, indicating the characteristic n-type semiconductor behavior of the passive films. They all display typical n-type semiconductor behavior, with their potential regions categorized into two distinct segments: a low-potential range (−2.5 to −1.0 V) and a high-potential range (−1.0 to −0.25 V). This potential division suggests the coexistence of dual donor density levels (N_d_) within the passive film structure. As the applied potential is raised to −0.75 V, the slope of the Mott–Schottky plot in the corresponding passive region progressively increases. This behavior is ascribed to an electronic structure transition within the passive film, corresponding to a change from low-potential to high-potential conditions. After that, the N_d_ and E_FB_ of the three HEA surface passive films were determined through Mott–Schottky curve fitting analysis, with the corresponding numerical values annotated in the figure. Notably, the HEAs with varying Cu content exhibited no statistically significant differences in their flat band potentials, while the N_d_ has an apparent difference. To investigate the influence of Cu content on passive film properties, the donor density (N_d_) of the HEAs was comparatively analyzed. The observed variation in N_d_ across compositions is linked to the film’s semiconductor behavior, where electrical conduction primarily occurs via charge transport mediated by cation and anion vacancies. In contrast, semiconducting passive films comprising metal oxides contain both anion and cation vacancies, and Cl^−^ compromises the oxide layer by occupying these defect sites. An increase in the donor density (N_d_) of the passive film is therefore accompanied by a higher concentration of charge-compensating vacancy defects, thereby enhancing the film’s electrical conductivity. This leads to a more pronounced E_FB_, which consequently reduces corrosion resistance. Among the series, the Cu_0.3_CoCrMoNi HEA exhibits the most stable passive film. This superior stability is indicated by its concurrently lower donor density (N_d_) and more negative flat-band potential (EFB) compared to the higher-Cu-content alloys.

### 3.4. XPS Result Analysis

**Figure 7:**
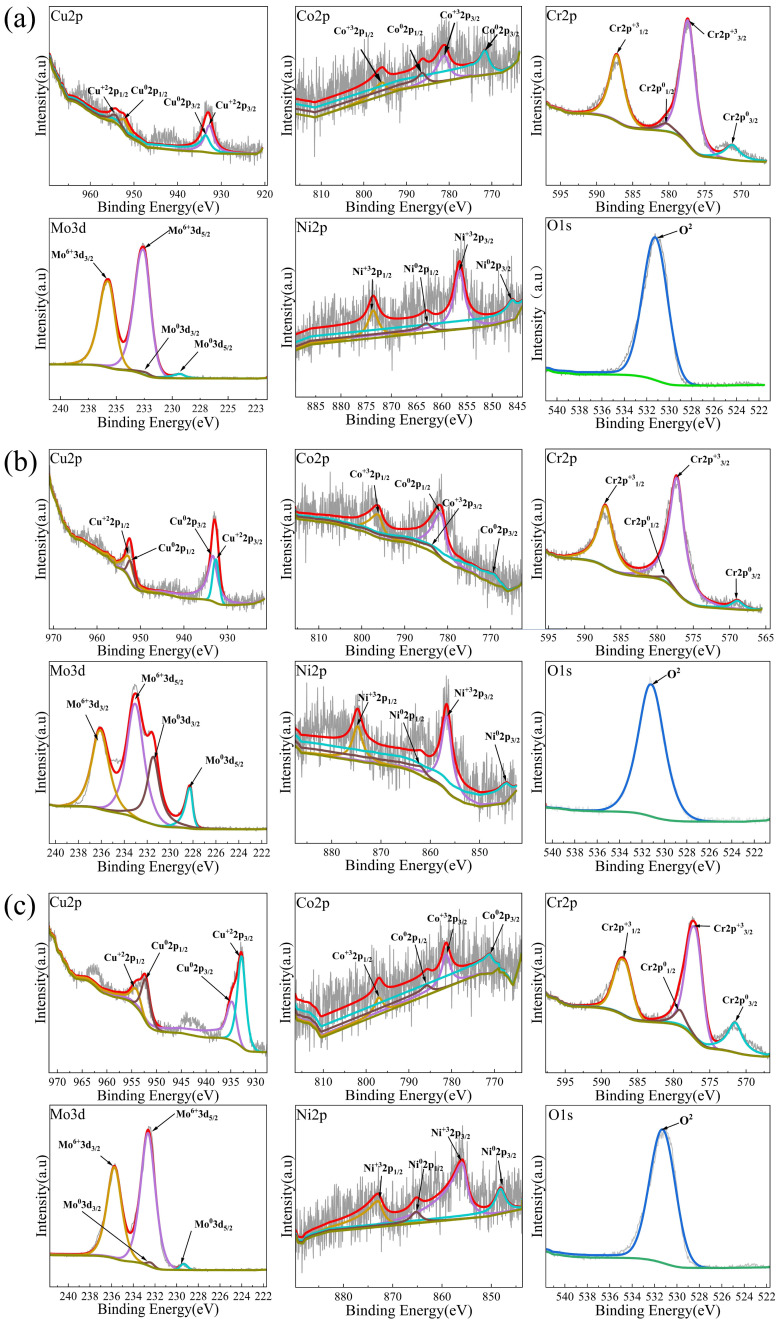
XPS patterns of passive films formed on Cu_X_CoCrMoNi: (**a**) Cu_0.3_CoCrMoNi, (**b**) Cu_0.6_CoCrMoNi, and (**c**) Cu_0.9_CoCrMoNi. The red line represents the fitted curve, the gray line represents the curve generated from the original data, and the green line represents the baseline.

Given that the chemical composition of the passive film largely governs the alloy’s corrosion resistance, this study therefore sought to elucidate the composition–property relationship, and the chemical states and elemental distribution within the passive films on Cu_x_CoCrMoNi HEAs were characterized using XPS. Figure 7 presents the XPS fine peak fitting results of Co2p, Cr2p, Mo3d, Ni2p, Cu2p, and O1s peaks resulting from the valence analysis of passive films on Cu_x_ (x = 0.3, 0.6, 0.9) CoCrMoNi HEA.

In the Cu_x_ (x = 0.3, 0.6, 0.9) CoCrMoNi HEA passive films, the Cu2p XPS spectrum can be resolved into two distinct components, i.e., Cu2p1/2 and Cu2p3/2, corresponding to metal oxide Cu^2+^ and metallic Cu, respectively. Notably, the Cu_0.3_CoCrMoNi sample exhibits a significantly lower Cu^2+^ concentration compared to the samples with higher Cu content (x = 0.6 and 0.9). The experimental evidence demonstrates that Cu_2_O additives deteriorate the passivation layer integrity [48], while the synergistic effect of Cl^−^ diffusion into the alloy matrix leads to the accelerated degradation of the HEA’s corrosion performance [49]. Similarity, the Cr 2p spectrum splits into two characteristic peaks, Cr 2p1/2 and Cr 2p3/2, corresponding to Cr^0^ and Cr^3+^ species. Notably, the Cu_0.3_CoCrMoNi alloy exhibits a significantly higher Cr_2_O_3_ content than comparative samples, as evidenced by the enhanced Cr^3+^ component. The enrichment of Cr_2_O_3_ significantly enhances the corrosion resistance of the passive film, by virtue of its stabilizing effect on the protective oxide layer [50]. The analysis also resolves the Mo spectrum into two spin–orbit split components (Mo 3d5/2 and 3d3/2), where the predominant signal from oxidized Mo6^+^ species surpasses the metallic Mo_0_, demonstrating MoO_3_ phase predominance over elemental Mo in the passive film composition. The MoO_3_ can effectively inhibit pitting in HEA, thereby promoting the protective property of passivation film [51]. The O1s spectrum, in comparison, displays a distinct single peak (designated as O_2_) with a binding energy centered at 531.6 eV, characteristic of passive oxide film formation.

The passive films, identified by XPS as Cu/Cr/Mo oxides, derive their protective function primarily from Cr_2_O_3_. This oxide forms a dense, continuous barrier that provides dual protection: it isolates the substrate and blocks aggressive Cl^−^ ions, thereby critically enhancing the alloy’s corrosion resistance. As evidenced by the corresponding peaks, the Cu_0.3_CoCrMoNi alloy exhibits a higher Cr_2_O_3_ concentration in its passive film, indicating the enhanced structural stability of the oxide layer. The enhanced corrosion resistance correlates with compositional evolution: the elevated Cu content induces the progressive substitution of Cr(III) oxide with Cu(I) oxide, as evidenced by the inverse proportionality between Cr_2_O_3_ and Cu_2_O concentrations during compositional analysis. The deterioration of passive film integrity and increased structural defects in the HEA progressively compromise their corrosion resistance, as evidenced by the increased Cu oxides. In addition, the passive film exhibits rapid Cl^−^ absorption. However, the Cu_2_O component within this film demonstrates relatively weak binding capacity and density. This compositional flaw triggers preferential dissolution, which propagates damage and weakens the overall protective capability, leading to diminished corrosion resistance in the HEA series.

**Figure 8:**
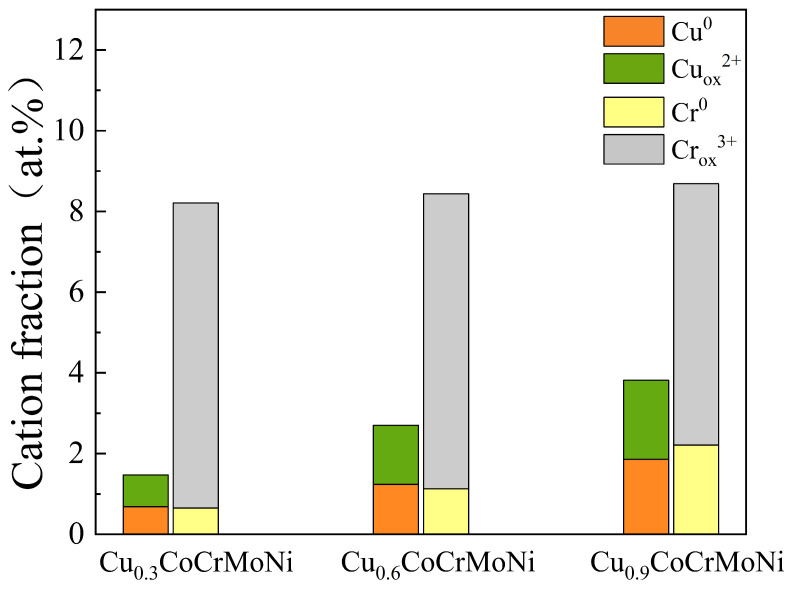
The comparison of the composition of Cu_x_ (x = 0.3, 0.6, 0.9) CoCrMoNi HEAs in passive films.

The results demonstrate that elevating Cu content in Cu_x_ (x = 0.3, 0.6, 0.9) CoCrMoNi HEAs significantly impacts their corrosion resistance. The phenomenon correlates with the observed phase segregation into distinct Cu-rich and Cu-poor regions, as clearly evidenced by the microstructural characterization data presented in Figure 2. As shown in Figure 3, all alloys developed stable passive zones, where the formation of dense passive films effectively arrested the progression of corrosion. To elucidate the underlying mechanism by which Cu content governs the corrosion resistance of the HEA passive film, an examination of Figure 5 and Figure 6 demonstrates that elevated Cu content (x = 0.3, 0.6, 0.9) in Cu_x_CoCrMoNi HEAs significantly enhances charge carrier density within the passive film. This phenomenon indicates enhanced charge penetration through the inherent defects of film, leading to accelerated substrate degradation through defect-mediated corrosion pathways. Higher Cu content favors the formation of Cu-rich phases, resulting in a corrosion product film dominated by less-protective copper oxides. The porous and loose structure of Cu oxide not only degrades the film quality directly but also suppresses the formation of protective Cr_2_O_3_. Consequently, the overall protectiveness diminishes with higher Cu content, a trend clearly confirmed by the decreasing Cr_2_O_3_ fraction shown in Figure 8. Therefore, as Cu content increases, Cu_2_O becomes a more prominent component within the passive film, particularly on the Cu-rich phases.

As Cu content increases, the passive film becomes increasingly enriched in copper oxides. The competitive suppression of Cr oxide formation by copper oxides degrades the film’s protectiveness against Cl^−^. Consequently, optimizing the composition by lowering Cu content is key to enhancing the corrosion resistance of the CuCoCrMoNi HEA.

## 4. Conclusions

A systematic investigation was conducted on the influence of Cu content (x = 0.3, 0.6, 0.9) on the microstructure and corrosion behavior of Cu_x_CoCrMoNi HEAs in 3.5 wt.% NaCl solution. The key findings are summarized below:

1. The phase structure of Cu_x_ (x = 0.3, 0.6, 0.9) CoCrMoNi HEAs encompasses a dual-phase structure comprising FCC1 and FCC2. The FCC1 phase is characterized by its Cu-rich composition, and the FCC2 phase is defined by its Cu-poor composition. The elements Co and Ni exhibit an abundance distribution within the Cu-rich phase.
2. Polarization and impedance tests show that reducing Cu content leads to a lower corrosion current density and a higher activation–passivation potential, indicating enhanced passivation ability, and a larger radius of the capacitive semicircle. This suggests that the Cu_0.3_CoCrMoNi HEA possesses superior corrosion resistance.
3. According to the analysis of the M-S curves of the Cu_x_ (x = 0.3, 0.6, 0.9) CoCrMoNi HEAs in a 3.5 wt.% NaCl solution, it is shown that the passive films on these alloys exhibit characteristics of n-type semiconductors. As the content of Cu increases, the corresponding values of N_d_ also rise, indicating that the charge density of the passive film increases, leading to a reduction in the stability of these films.
4. XPS analysis reveals that the composition of the passive films on Cu_x_ (x = 0.3, 0.6, 0.9) CoCrMoNi HEAs is mainly Cr_2_O_3_ and Cu_2_O. However, a higher composition of Cu_2_O is observed in the passive film of the HEA with increased Cu content, which disrupts the uniformity of the dense Cr_2_O_3_, leading to a decrease in the corrosion resistance of the HEA. Therefore, the corrosion resistance of the Cu_x_ (x = 0.3, 0.6, 0.9) CoCrMoNi HEAs decreases as Cu increases.

## Figures and Tables

**Table 1:** Atomic ratios of elements in Cu_x_ (x = 0.3, 0.6, 0.9) CoCrMoNi HEA tested by EDS (at%).

Alloy	Cu	Co	Cr	Mo	Ni
Cu_0.3_CoCrMoNi	6.96	26.63	23.26	23.26	23.26
Cu_0.6_CoCrMoNi	13.04	21.74	21.74	21.74	21.74
Cu_0.9_CoCrMoNi	18.36	20.41	20.41	20.41	20.41

**Table 2:** Composition of point elements in Cu_x_ (x = 0.3, 0.6, 0.9) CoCrMoNi HEA (at%).

Alloy	Point	(at%)				
		Cu	Co	Cr	Mo	Ni
Cu_0.3_CoCrMoNi	A	2.59	27.20	27.26	22.07	20.89
	B	9.73	27.23	20.97	8.52	33.54
	A	3.17	26.35	26.99	22.07	21.42
	B	9.05	27.77	21.20	7.93	34.05
Cu_0.6_CoCrMoNi	A	3.15	27.14	26.78	22.07	20.89
	B	9.27	28.13	21.26	8.07	33.28
	A	2.64	27.30	27.05	21.49	21.52
	B	8.90	27.93	22.09	8.15	32.93
Cu_0.9_CoCrMoNi	A	2.08	27.24	27.20	23.80	19.68
	B	8.85	28.43	21.47	8.34	32.90
	A	2.89	27.36	27.03	22.66	20.05
	B	8.67	28.15	21.62	8.42	33.14

**Table 3:** Potentiodynamic polarization parameters of Cu_x_ (x = 0.3, 0.6, 0.9) CoCrMoNi HEA in 3.5 wt.% NaCl solution.

Alloy	I_corr_	E_pp_	E_b_	I_pass_
Cu_0.3_CoCrMoNi	2.138 × 10^−7^	−0.77533	−0.0512	5.58 × 10^−5^
Cu_0.6_CoCrMoNi	5.0647 × 10^−7^	−0.91735	−0.0645	6.26 × 10^−5^
Cu_0.9_CoCrMoNi	1.8989 × 10^−6^	−0.91167	−0.0515	8.09 × 10^−5^
SS316	6.2 × 10^−7^		+0.41	5.75× 10^−6^

**Table 4:** EIS parameters of Cu_x_ (x = 0.3, 0.6, 0.9) CoCrMoNi HEA in 3.5 wt.% NaCl solution.

Alloy	R_s_ (Ω·cm^2^)	R_ct_ (Ω·cm^2^)	Constant Phase Elements		
			Y_0_	n	χ^2^
Cu_0.3_CoCrMoNi	11.82 ± 0.02	182.6 ± 4	3.5957 × 10^−4^ ± 0.04	0.74878 ± 0.003	3.03 × 10^−3^
Cu_0.6_CoCrMoNi	12.31 ± 0.03	81.86 ± 2	2.5581 × 10^−4^ ± 0.03	0.78782 ± 0.004	2.68 × 10^−3^
Cu_0.9_CoCrMoNi	11.72 ± 0.03	42.34 ± 2	2.6325 × 10^−4^ ± 0.03	0.80581 ± 0.003	2.92 × 10^−3^

## Data Availability

The original contributions presented in this study are included in the article. Further inquiries can be directed to the corresponding author.
